# Knee and Ankle Joint Angles Influence the Plantarflexion Torque of the Gastrocnemius

**DOI:** 10.14740/jocmr2107w

**Published:** 2015-06-09

**Authors:** Dennis Landin, Melissa Thompson, Meghan Reid

**Affiliations:** a School of Kinesiology, Louisiana State University, Baton Rouge, LA, USA

**Keywords:** Gastrocnemius, Plantarflexion, Joint angles, Torque

## Abstract

**Background:**

The gastrocnemius (GA) is the lone bi-articular muscle of the leg, crossing both the knee and ankle. As with any bi-articular muscle, both joints affect its length/tension curve. The role of the GA as a plantarflexor is firmly established; however, no current research has investigated how changes in knee and ankle joint positions on its ability to generate a plantarflexion (PF) torque. This paper reports on the PF force generated by the GA at specific knee and ankle joint combinations.

**Methods:**

The right GA of 26 participants was electrically stimulated via surface electrodes following a standardized protocol at 24 knee and ankle joint combinations. Three stimulations were applied at each of the 24 positions. Data were recorded on three dependent measures: the passive moment, which was the PF moment created by the tissue without stimulation, the maximum moment, which was the highest PF moment during the stimulation and included the passive moment, and the stimulated moment, which reflected the PF moment during stimulation minus the passive moment.

**Results:**

A straight knee and dorsiflexed ankle create the position in which the GA generates the greatest PF moment, but it is also the position of greatest length. This finding is in contrast to conclusions from previous research with bi-articular muscles, which has consistently shown that the greatest length is not a muscle’s optimal length. The full ranges of motion for the knee and ankle apparently do not elongate the GA beyond its optimal length for producing a PF moment. Clinicians commonly evaluate GA status with the patient seated and the foot subject to gravity.

**Conclusions:**

The present results indicate that manual testing of the GA in isolation should be performed, whenever possible, with the knee extended and the ankle dorsiflexed to potentially elicit the maximum PF torque from the GA.

## Introduction

The gastrocnemius (GA) is the lone bi-articular muscle of the leg, crossing both the knee and ankle. It is a fusiform, two-headed muscle with nearly a vertical orientation of its fascicles. The fascicular arrangement allows the GA to contribute to the rapid and explosive movements seen in running and jumping [1]. Its proximal attachments are comprised of medial and lateral heads, which arise from the popliteal surface of the femur superior to the medial and lateral femoral condyles, respectively. The bellies of the two heads sit relatively high on the posterior leg and then join with soleus to form the calcaneal (Achilles) tendon that inserts on the posterior calcaneus. When considered in conjunction with the deeper soleus muscle, the two are collectively referred to as the triceps surae. These two muscles provide approximately 80% of force of plantarflexion (PF), which is a principal component to a large portion of the gait cycle and essential to nearly all forms of human locomotion. PF forces can be quite high. It is estimated that young males can generate PF torque ranging between 1,000 and 1,780 N [2].

General details regarding the actions of the GA, particularly in PF, have been known for well over a century [3]. Despite this long history, its actions have received some recent attention. Di Nardo et al [4] used sEMG to record the activation patterns of the GA while walking. Healthy young adults walked for 5 min at a self-selected speed while sEMG data were collected on the tibialis anterior and the GA lateralis. Their main findings showed that the GA lateralis was active in the stance phase during the transition from flat foot contact to toe-off, and then again in the final swing phase. However, another pattern was also revealed. In the majority of the strides measured (about 78%), the GA lateralis was silent in the pre-swing phase, but in about 22% of the strides the muscle was also active in pre-swing.

The bi-articular design of the GA muscle allows it to flex the knee in addition to plantarflexing the ankle [3, 5-7]. As with any bi-articular muscle, both joints affect its length/tension curve [2], thereby producing favorable and unfavorable joint angle combinations. Two other recent studies have investigated how various knee and ankle positions influenced the actions of the GA.

Riemann et al [8] investigated the GA’s contribution to the ankle joint’s stability across three ankle angles. More specifically, these investigators measured the stiffness values of the GA as the subject’s ankles were passively moved from 10° of dorsiflexion through the neutral position and into 10° of PF, while the knee was positioned at 0° and 90°. The results revealed that the 0° knee angle produced higher stiffness values across the three ankle positions than the 90° knee angle. This shows that both joints influence the forces the GA produces. The stiffness index increased as the ankle moved from PF to dorsiflexion and actually began early in the neutral position.

The role of the GA as a knee flexor, a function about which little is known, was explored by Li et al [9]. These investigators studied various combinations of the knee and ankle joint angles to determine which combination enabled the GA to produce the largest knee flexion moment. The main findings showed a significant interaction between knee and ankle joint angles. Polynomial trend analyses revealed that the effects of both joints were linear. The greatest knee flexion moment for the GA occurred when the knee was at 0° (anatomical position) across all ankle joint angles (Li et al (2002) labeled the knee angles from the anatomical position as follows: 180°, 165°, 150°, 135°, 120°, 105°, 90°, and 75°. We used the more common clinical sequence starting with 0° for the anatomical position, and then progressing 15°, 30°, 45°, 60°, 75°, 90°, and 105° of knee flexion. We converted the Li et al sequence to ours to avoid confusion). The largest reduction in the GA knee flexion moment occurred when the knee position moved from 0° to 15° and then decreased linearly from that point. When the knee angle reached 90° and less, very little knee flexion moment was recorded. The results from Li et al show that the GA can play a substantial role in knee joint stability provided the knee is not flexed beyond 15°.

These studies highlight the importance of considering both joint angle positions when looking at the function of the GA. The role of the GA as a plantarflexor is firmly established; however, no current research has investigated the effect of changing both ankle and knee joint positions on the PF moment. As these joint angles are altered, muscle force production factors and moment arm length also change. The force a muscle produces depends on the amount of stimulation it receives, its length at the moment of stimulation, and its contraction velocity [10, 11]. While changes in joint angles do not determine contraction velocity and level of stimulation, a muscle’s length is greatly affected by such changes. Alterations in joint angles affect both the moment arm and length of the muscle [12], which may result in significant changes to the PF moment of the GA.

Therefore, the current project focused on the PF force generated by the GA at specific knee and ankle joint combinations. The goal was to identify the optimal combination for the GA to generate a PF moment. It was hypothesized that the PF moment of the GA would increase as both knee flexion and PF decreased. It was also of interest to learn if the optimal joint angle combinations for a PF moment were similar to the most advantageous joint positions reported previously [8, 9].

## Method

### Participants

Twenty-six undergraduate female students (age: 19.3 ± 0.9 years; mass: 54.2 ± 4.2 kg; height: 165.5 ± 5.7 cm) participated in this project. Several inclusion criteria were used during the recruitment process. First, all subjects needed a relatively lean physique. This ensured that the removable casts used to control the knee joint angles would fit properly. The second inclusion criterion was no history of lower extremity injury or a physical abnormality. This information was obtained by questioning each potential participant during the initial visit to the laboratory. Thirdly, all responses to items on the physical activity readiness quotient [13] had to be negative. When a prospective participant met these criteria, the next step was to read and sign the informed consent document approved by the University’s Institutional Review Board.

### Equipment

A Biodex System III Dynamometer (Biodex Medical Systems, Shirley, NY) controlled the angle of the subject’s right ankles and measured the isometric PF torque (Nm). Calibration checks, performed at regular intervals on this unit, ensured the accuracy of its measurements. Contraction of the GA was induced through surface electrodes with a grass electrical stimulation generator (model SD9B). Electrodes were placed across the common belly of the medial and lateral heads of the GA. The knee joint angle was controlled with removable casts in eight positions (0° the anatomical position, 15°, 30°, 45°, 60°, 75°, 90°, and 105°) during testing.

### Procedures

Participants were positioned in the Biodex with the right ankle joint parallel to the rotational axis of the dynamometer. Electrodes were placed on the GA across the belly of the muscle just distal to the point where the two heads merge. The amount of electrical stimulation was standardized across the subjects using the formula and procedure first devised by Li et al [9], and then used in subsequent work with the biceps brachii, triceps brachii, and rectus femoris [14, 15].

The magnitude of 15 Hz train square wave stimulation with 10 ms pulse duration was gradually increased from 0 V. The voltage that produced a PF moment equal to 1.7% of body weight, multiplied by stature, and held for 5 s, was designated as the testing voltage. This formula ensured that the voltage applied, while variable across each individual, produced the same level of stimulation in each subject. Pilot testing revealed that this level of stimulation was both effective and reasonably comfortable for the subjects.

The testing order was randomized for each subject. The PF moment for each joint angle combination was recorded before, during, and after stimulation and tabulated as three dependent measures. First, was the passive moment (PM), which was the PF moment created by the tissue without stimulation and was obtained by taking the mean of the PF moment before and after the stimulation. This was expected to change as the knee and ankle were moved through the various positions, as these joint angles change muscle and connective tissues undergo passive length changes that produce the PM. The second dependent measure was the maximum moment (MM), which was the highest PF moment during the stimulation, and included the PM. The third measure was the stimulated moment (SM), which reflected the PF moment produced by the stimulation minus the PM contribution and represented the difference between PM and MM. Across all trials, the subjects were instructed to neither assist nor resist the contraction, and remain as still as possible.

Eight knee flexion angles (0°, 15°, 30°, 45°, 60°, 75°, 90°, and 105°) and three ankle angles (-15° dorsiflexion, 0°, +15° PF) created 24 joint combinations. Knee angles were maintained with removable casts, while ankle angles were controlled by the dynamometer. At each position the right GA was electrically stimulated, in a counterbalanced order, three times via surface electrodes.

### Analysis

Data for the dependent measures were compiled by averaging across the three trials at each joint combination. These values were averaged into a single score for each dependent measure at each joint combination and then analyzed with a two factor (knee × ankle) within-subject ANOVA with repeated measures. *Post hoc* polynomial trend analyses and Turkey’s HSD were applied as needed. The alpha level was set at 0.05, and adjusted with the Bonferroni procedure if necessary.

## Results

The stimulation range across the participants was 75 - 100 V. None of the participants experienced skin damage; however, several participants did report some lingering soreness in the hours immediately following the test.

Maximum moment values appear in Table 1.

**Table 1:** Plantarflexion Maximum Moment in Nm (SD)

Knee angle (°)	Ankle angle (°)			
	-15 pf	0	+15 df	Mean
0	20.5	21.11	24.95	22.18
15	18.91	20.35	21.43	20.23
30	17.65	20.47	23.72	20.61
45	18.96	20.30	22.41	20.55
60	16.99	18.5	20.71	18.73
75	14.93	17.51	20.9	17.78
90	15.03	18.11	20.11	17.75
105	13.02	14.95	18.17	15.38
Mean	16.99	18.91	21.55	

Differences greater than 7.61 between knee/ankle joint combinations are significant (P = 0.05). pf: plantarflexed position; df: dorsiflexed position.

Significant main effects were found for the knee F(7,203) = 11.24, P = 0.001 and ankle F(2,58) = 48.75, P = 0.001. Follow-up polynomial contrasts revealed both effects were linear F(1,29) = 37.35, P = 0.001 and F(1,29) = 75.01, P = 0.001 for the knee and ankle, respectively. The knee × ankle interaction was not significant (P = 0.279). As knee flexion increased and the ankle was plantarflexed the maximum moment decreased. The greatest torque (24.95 ± 10.1 Nm) was obtained with the knee at 0° and the ankle dorsiflexed +15°. The lowest torque (13.02 ± 4.9 Nm) was obtained with the knee flexed to 105° and the ankle plantarflexed -15°. Tukey’s HSD *post hoc* tests revealed these values to be significantly different (P = 0.05).

Table 2 displays the values for the PF moment in the passive tissue condition.

**Table 2:** Plantarflexion Passive Moment in Nm (SD)

Knee angle (°)	Ankle angle (°)			
	-15 pf	0	+15 df	Mean
0	12.63	15.13	20.69	16.15
15	11.38	14.10	17.33	14.27
30	9.68	14.01	18.88	14.19
45	9.80	12.01	16.65	12.82
60	9.02	11.78	15.57	12.12
75	8.46	11.36	15.39	11.73
90	7.77	10.63	15.08	11.16
105	5.75	9.52	13.99	9.75
Mean	9.31	12.31	16.69	

Differences greater than 9.15 between knee/ankle joint combinations are significant (P = 0.05). pf: plantarflexed position; df: dorsiflexed position.

Mauchly’s test of sphericity was significant for the knee, ankle, and the interaction for this dependent measure. Consequently, the Huynh-Feldt correction was used for the PM results. Significant main effects were obtained for the knee F(4.14,120.19) = 20.13, P = 0.001 and for the ankle F(2,36.27) = 215.93, P = 0.001. Follow-up polynomial contrasts showed a significant linear effect of the knee F(1,29) = 95.9, P = 0.001, while the effect of the ankle was linear F(1,29) = 239.08, P = 0.001 and quadratic F(1,29) = 22.58, P = 0.001. The knee × ankle interaction was not significant (P = 0.226). The greatest torque (20.69 ± 10.1) was recorded with the knee at 0° and the ankle dorsiflexed +15°. The 105/-15 knee and ankle combination produced the smallest PF moment (5.75 ± 3.2). Tukey’s HSD post hoc tests revealed these differences to be significant (P = 0.05).

The results for the SM are displayed in Table 3.

**Table 3:** Plantarflexion Stimulated Moment in Nm (SD)

Knee angle (°)	Ankle angle (°)			
	-15 pf	0	+15 df	Mean
0	4.01	6.0	7.92	5.97
15	4.0	5.99	7.28	5.75
30	4.99	6.48	7.99	6.48
45	5.65	8.32	8.98	7.65
60	5.03	6.56	7.93	6.50
75	5.46	6.32	6.46	6.08
90	5.00	7.44	7.18	6.54
105	4.25	5.49	6.88	5.54
Mean	4.79	6.57	7.57	

Differences greater than 4.73 between knee/ankle joint combinations are significant (P = 0.05). pf: plantarflexed position; df: dorsiflexed position.

Mauchly’s test for sphericity was significant for the knee and the knee × ankle interaction for this dependent measure. The Huynh-Feldt correction was used for these results. The assumption for sphericity was met for the ankle. The effect of the ankle was significant for the SM F(2, 58) = 18.12, P = 0.001. Follow-up polynomial contrasts revealed that this effect was linear F(1, 29) = 25.16, P = 0.001. The greatest torque (8.98 ± 4.3) occurred with the knee at 45° and ankle dorsiflexed at +15°. The lowest torque (4.0+3.1) was recorded at the 15° knee and the -15° plantarflexed ankle position. Tukey’s HSD *post hoc* tests revealed these differences to be significant (P < 0.05). Neither the effect of the knee nor the knee × ankle interaction was significant.

## Discussion

The PF function of the GA has been recognized for over a century. This project sought to further define this role by investigating how various combinations of knee and ankle joint angles influence the PF moment produced by the GA. The principal finding regarding the MM was that a fully extended knee (0°) and a dorsiflexed ankle (+15°) created the highest (24.95 ± 10.1 Nm) torque values, while the 105° knee and a plantarflexed ankle (-15°) produced the smallest values (13.02 ± 4.9 Nm). This reduction in Nm was linear. We used the same knee and ankle combinations (Li et al (2002) labeled the knee angles from the anatomical position as follows: 180°, 165°, 150°, 135°, 120°, 105°, 90°, and 75°. We used the more common clinical sequence starting with 0° for the anatomical position, and then progressing 15°, 30°, 45°, 60°, 75°, 90°, and 105° of knee flexion. We converted the Li et al sequence to ours to avoid confusion) as Li et al [9]. They reported that the GA produced the greatest knee flexion moment in the same joint combination, although the force (Nm) was not as high (approximately 9.5 Nm), as we noted for PF. We contend that this is most likely due to the disparity in the mechanical advantage of the knee and ankle lever systems. At the knee, the GA works in a third class lever, which produces little mechanical advantage. At the ankle, however, the GA is part of a second class lever, which has considerable mechanical power. Based on our results, and those of Li et al [9], it is the 0° knee position and a dorsiflexed ankle that creates the optimal length for the GA to work at either joint. It is apparently the mechanical advantage of the lever system that ultimately determines the GA’s joint flexion forces.

Regarding the lowest torque, these results are somewhat in contrast to Li et al [9]. Their results showed that flexing the knee to 90° or more produced insignificant further decreases in torque regardless of the ankle position. More specifically, the knee flexion moment did not significantly decrease as the knee was flexed to 105° with the ankle plantarflexed, neutral, or dorsiflexed. We reported that the PF joint moment decreased linearly from the 0/+15 joint combination, through the 105/-15 combination, at which point the GA was in its most shortened position. The fact that Li et al found the smallest knee flexion moment before reaching the shortest position for the GA may be due to the third class lever at the knee. The mechanical properties of a third class lever may render a muscle insufficient before it reaches its shortest length. The second class lever of the ankle, however, causes continual declines in the GA PF moment. Further research, using knee flexion angles less than 105° may provide further information on this issue.

Riemann et al [8] investigated the GA’s contribution to the stability of the ankle joint complex across three ankle angles, but did not employ external stimulation nor were their subjects performing voluntary muscle contractions. Therefore, the Reimann et al protocol resembles our PM condition. These investigators measured the stiffness values of the GA as the subject’s ankles were moved from 10° of dorsiflexion through the neutral position and into 10° of PF, while the knee was positioned at either 0° or 90°. Their results revealed that the 0° knee angle produced higher stiffness values and that these values increased as the ankle moved from PF to dorsiflexion. Furthermore, the increase in the stability of the ankle joint complex began early in the neutral position. Our PM results are consistent with those of Reimann et al [8]. We found the greatest PM in the 0°/+15° joint combination, and the torque in that position was quite high (20.69 ± 10.1). This value represented over 75% of the MM. Clearly the passive tissues create a substantial force when the knee and ankle are in the 0°/+15° position. While Reimann et al [8] used only two knee angles, compared with eight used in the present study, the main results are congruent. We found that the PM increased as the knee moved into a more extended position and the ankle was dorsiflexed. As the GA was moved into its most elongated position, the stability of the joint complex and the PM increased.

The SM was defined as the MM minus the PM. It accounted for a relatively small percentage of the MM. The ankle was the only significant factor in this measure, and its effect was linear showing that moving from -15° of PF to +15° of dorsiflexion resulted in an increase in the SM. The highest SM occurred at the 45°/+15° knee and ankle position, while the lowest was obtained at the 15°/-15° knee/ankle position. The difference in SM between these two positions was about 5 Nm (P < 0.05). We expected the SM values to follow the same linear pattern seen in the MM and PM data, so these results are not easily understood. While neither the knee nor the knee × ankle interaction was significant, it is interesting to observe how the SM changed across the knee angles. As Table 3 displays, knee angles from 30° to 90° led to the highest SM recordings across all ankle positions, with the 45° knee angle being the most favorable. The joint combinations which differed significantly from the 45°/+15° combination involved PF and the knee angles of 0°, 15°, and 105°. When the effect of the passive tissue was removed, the GA generated the largest SM when the muscle was not at its maximum length. This finding with SM data has not been noted in our previous work [9, 14, 15] and needs further investigation.

### Clinical application

Clinicians should be aware that having a patient seated at the end of an examination table with the foot subject to gravity, while useful for evaluating the triceps surae, is a poor position from which to assess the GA as a plantarflexor. This position places the knee beyond 60° of flexion and maintains a somewhat plantarflexed foot, both of which lead to significant declines in GA torque production. Even if the clinician holds the patients in a dorsiflexed position, the flexed knee will still mask the GA’s true strength. Manual testing of the GA in isolation should be performed, whenever possible, with the knee extended and the ankle dorsiflexed to potentially elicit the maximum PF torque from the GA.

### Conclusions

A straight knee and dorsiflexed ankle create the position in which the GA generates the greatest PF moment, but it is also the position of greatest length. This finding is in contrast to conclusions from previous research with bi-articular muscles, which has consistently shown that the greatest length is not a muscle’s optimal length. The full ranges of motion for the knee and ankle apparently do not elongate the GA beyond its optimal length for producing a PF moment.
